# Homeostatic NREM sleep and salience network function in adult mice exposed to ethanol during development

**DOI:** 10.3389/fnins.2023.1267542

**Published:** 2023-11-14

**Authors:** Prachi Shah, Aayush Kaneria, Gloria Fleming, Colin R. O. Williams, Regina M. Sullivan, Christian H. Lemon, John Smiley, Mariko Saito, Donald A. Wilson

**Affiliations:** ^1^Emotional Brain Institute, Nathan Kline Institute for Psychiatric Research, Orangeburg, NY,United States; ^2^School of Biological Sciences, University of Oklahoma, Norman, OK, United States; ^3^Department of Child and Adolescent Psychiatry, NYU School of Medicine, New York, NY, United States; ^4^Division of Neurochemistry, Nathan Kline Institute for Psychiatric Research, Orangeburg, NY,United States; ^5^Department of Psychiatry, New York University Medical Center, New York, NY,United States

**Keywords:** fetal alcohol spectrum disorder, functional connectivity, salience network, sleep homeostasis, insomnia

## Abstract

Developmental exposure to ethanol is a leading cause of cognitive, emotional and behavioral problems, with fetal alcohol spectrum disorder (FASD) affecting more than 1:100 children. Recently, comorbid sleep deficits have been highlighted in these disorders, with sleep repair a potential therapeutic target. Animal models of FASD have shown non-REM (NREM) sleep fragmentation and slow-wave oscillation impairments that predict cognitive performance. Here we use a mouse model of perinatal ethanol exposure to explore whether reduced sleep pressure may contribute to impaired NREM sleep, and compare the function of a brain network reported to be impacted by insomnia–the Salience network–in developmental ethanol-exposed mice with sleep-deprived, saline controls. Mice were exposed to ethanol or saline on postnatal day 7 (P7) and allowed to mature to adulthood for testing. At P90, telemetered cortical recordings were made for assessment of NREM sleep in home cage before and after 4 h of sleep deprivation to assess basal NREM sleep and homeostatic NREM sleep response. To assess Salience network functional connectivity, mice were exposed to the 4 h sleep deprivation period or left alone, then immediately sacrificed for immunohistochemical analysis of c-Fos expression. The results show that developmental ethanol severely impairs both normal rebound NREM sleep and sleep deprivation induced increases in slow-wave activity, consistent with reduced sleep pressure. Furthermore, the Salience network connectome in rested, ethanol-exposed mice was most similar to that of sleep-deprived, saline control mice, suggesting a sleep deprivation-like state of Salience network function after developmental ethanol even without sleep deprivation.

## Introduction

Adverse events during development can have significant consequences for emergence of physical and mental health issues in later life (McEwen, 2003; Barker, 2004; Gluckman and Hanson, 2004; Nemeroff, 2004; Green et al., 2010; Perry and Sullivan, 2014; Rincon-Cortes and Sullivan, 2014; Padmanabhan et al., 2016). Mechanisms linking early life events with later life outcomes include physiological, anatomical, hormonal, epigenetic and behavioral processes (Heim et al., 1997; Blaze et al., 2015; Moreno-Lopez et al., 2020; Roos et al., 2021). One additional potential intermediary between early adverse events and later life outcomes that has gained recent attention is sleep impairment. Adverse events during early life ranging from trauma (Bader et al., 2007; Insana et al., 2012; Lewin et al., 2019) to toxin exposure (Criado et al., 2008; Troese et al., 2008; Pesonen et al., 2009; Chen et al., 2012; Raineki et al., 2017; Inkelis and Thomas, 2018; Ipsiroglu et al., 2019; Jansen et al., 2019) can induce life-long sleep impairment that has been shown to correlate with and/or statistically mediate adult behavioral outcomes (Quach et al., 2009; Petrov et al., 2016; Wilson et al., 2016; Lewin et al., 2019; Bian et al., 2022; Hosokawa et al., 2022). Sleep plays an important role across the lifespan in memory, cognition and emotional regulation–domains overlapping with those impacted by early adverse events–with impairments in all observed after sleep restriction (Carskadon, 2011; Fairholme and Manber, 2015; Jansen et al., 2019; Muehlroth et al., 2020).

While acute and chronic ethanol exposure can induce changes in both NREM and REM sleep in adolescents and adults (Landolt et al., 1996; Ehlers and Slawecki, 2000; Ehlers and Criado, 2010; Thakkar et al., 2015; Abrahao et al., 2020; Amodeo et al., 2020; Koob and Colrain, 2020), perinatal exposure can have life-long impacts on sleep. Fetal alcohol spectrum disorder (FASD) may affect more than 1:100 children in the United States (Lange et al., 2017; May et al., 2018; Popova et al., 2023). Humans with FASD, and animal models thereof, display reduced time asleep, NREM sleep fragmentation and reduced slow-wave activity that is expressed during childhood and extends into adulthood (Stone et al., 1996; Criado et al., 2008; Troese et al., 2008; Wengel et al., 2011; Chen et al., 2012; Goril et al., 2016; Wilson et al., 2016; Inkelis and Thomas, 2018; Lewin et al., 2018; Apuzzo et al., 2020; Chandler-Mather et al., 2021). According to current models of sleep the probability of sleep onset is modulated by two processes, circadian rhythms and sleep pressure which builds during waking (Borbely and Achermann, 1999). Developmental ethanol disrupts both circadian behavioral rhythms (Earnest et al., 2001) and oscillations in clock gene expression (Farnell et al., 2008), though impacts on sleep pressure are less well understood. Given the widespread neural and glial cell loss induced by developmental ethanol (Ikonomidou et al., 2000; Wozniak et al., 2004; Guizzetti et al., 2014; Saito et al., 2019; Smiley et al., 2019), sensing of sleep pressure could be impaired by early ethanol exposure.

One assay of sleep pressure is the homeostatic sleep response (e.g., sleep rebound) to periods of sleep deprivation. Astrocytes play a critical role in homeostatic sleep (Halassa et al., 2009) and are reduced in number and function by developmental ethanol (Valles et al., 1996; Rubert et al., 2006; Trindade et al., 2016). If sleep is impaired transiently or chronically by ethanol exposure, and sleep homeostasis is also impaired, this may exacerbate the effects of the original sleep loss by preventing rebound sleep. In fact, impaired sleep homeostasis has been linked to cognitive impairment (Boly et al., 2017).

Here we used sleep deprivation induced by environmental enrichment and gentle handling to address two questions. First, is sleep homeostasis following imposed sleep deprivation affected by developmental ethanol exposure? Second can sleep depriving adults exposed to developmental saline induce changes in network functional connectivity that mimic that observed in mice with developmental ethanol-induced sleep disruption? For the second question we focused on the Salience network. The Salience network is composed of insula cortex, prefrontal and limbic regions and responds to biologically or emotionally salient external or internal events such as rewards or dangers, and has been described as involved in the perception and response to homeostatic demands (Seeley, 2019). The Salience network has been described in both humans and rodents (Seeley et al., 2007; Seeley, 2019). Insomnia has been associated with changes in intrinsic Salience network connectivity (Khazaie et al., 2017), and in connectivity between Salience network and other networks such as the Default Mode Network (Khazaie et al., 2017; Li et al., 2018; Chee and Zhou, 2019). Furthermore, FASD has been reported to impact Salience network function (Little et al., 2018), as well as other networks (Santhanam et al., 2011; Roussotte et al., 2012; Donald et al., 2016; Wozniak et al., 2017). We predicted that sleep homeostasis may be impaired in adult mice exposed to developmental ethanol, in addition to changes in baseline sleep, and inducing a chronic sleep deprived connectivity pattern in the Salience network which could contribute to behavioral impairments.

## Methods

### Subjects

C57BL/6 J male and female mice were used. At P7, pups were given injections either containing saline (male *n* = 18; female n = 19) or 2.5 g/kg ethanol (male *n* = 19; female n = 21), administered subcutaneously using a well-characterized fetal alcohol binge exposure protocol (Ikonomidou et al., 2000; Saito et al., 2007). P7 ethanol exposure is a mouse model of human third trimester binge exposure (Olney et al., 2002) and occurs during a period of rapid brain growth and synaptogenesis (Dobbing and Sands, 1979; Rice and Barone, 2000). Two injections were given, separated by a 2-h interval, which has been previously established to produce reliable blood ethanol concentrations between 0.35 and 0.45 g/dL that persisted for several hours (Saito et al., 2007). The pups were then returned to their home cage and litter, weaned between P25-30, and then housed (cage width x length x height:18 x 28 × 12 cm) with same sex littermates until data collection at P90 ± 10 days. A 12:12 light dark cycle was used and food and water were available *ad lib.* Data collection began at P90. As previously reported (Wozniak et al., 2004; Wilson et al., 2016), body weight did not vary between saline and ethanol-exposed adult mice [saline group mean (± SEM) = 23.7 ± 1.7 g (females = 21,4 ± 1.0 g; males = 29.5 ± 0.5 g); ethanol group mean = 24.9 ± 1.5 g (females = 21.7 ± 0.88 g; males = 28.0 ± 0.6 g)]. All procedures were carried out with the approval of the Nathan Kline Institute IACUC and in compliance with NIH guidelines for proper animal treatment.

### Telemetry recording

Isoflurane was used to anesthetize adult mice, which were then placed in a stereotaxic apparatus and implanted with a single recording electrode in the frontal cortex (0–1 mm anterior Bregma, 1–2 mm lateral, 1 mm ventral) and a reference lead into the contralateral posterior cortex, as previously described (Wilson et al., 2016; Lewin et al., 2018). The local field potential (LFP) electrodes were linked to a single-channel telemetry device (DSI, model ETA-F10), which was implanted under the skin on the back. After the surgery, mice were allowed to recover for at least 3 days before baseline recording commenced. Mice were recorded in individual sound-attenuating chambers equipped with telemetry receivers. Recordings lasted between 3 and 5 days, with an initial 24 h period of baseline recording, followed by sleep deprivation and recovery protocols described below. All LFP recordings were captured at a rate of 1,000 Hz and analyzed in Spike2 software (Cambridge Electronic Design). Delta frequency (0.1–5 Hz) oscillations were used to identify the presence of slow-wave activity and NREM sleep. The low-pass filtered LFPs were used to extract the root mean square (r.m.s.) delta power. Epochs lasting 14 s with high delta power were identified if they were at least 1 standard deviation above the mean power during a 24-h period, and artifacts were removed before calculating the mean and standard deviation. With this method, the minimum NREM sleep or waking bout was a single 14 s epoch. In some animals, periods of LFP-identified NREM sleep were confirmed by visual inspection of animal behavior as previously described (Lewin et al., 2019). Given that EMG was not recorded, REM sleep was not assessed here.

### Sleep deprivation

Sleep deprivation was initiated during the first half of the light phase (lights on at 7 am, sleep deprivation onset at 10 am ± 1 h) and occurred in the animal’s home cage. Cage tops were removed and small plastic objects and/or scents (e.g., peppermint) were occasionally added and replaced in the cage as enrichment (Huber et al., 2007). An attempt was made to provide a standard sequence of enrichment across animals. Sleep/wake state was continually monitored both visually and with cortical LFP recordings. Toward the end of the 4 h session, some animals required a gentle touch to keep them awake. Two mice were excluded from the data set due to poor deprivation assessed post-hoc with LFP (they expressed >25% time in NREM sleep). Simultaneously with the deprivation protocol, littermate rested controls were maintained in their home cages in their separate sound attenuating chambers with LFP recording of sleep/wake state.

After 4 h of sleep deprivation or control, animals were given one of two manipulations. To assess NREM sleep homeostasis, enrichment stimuli were removed, cage tops replaced and the home cage returned to its sound attenuating chamber. Rested control mice were left undisturbed. Recordings were continued for at least 5 h to assess rebound NREM sleep and changes in slow-wave activity. Alternatively, to assess neural activity during sleep deprivation induced by environmental enrichment, animals were sacrificed immediately after the 4 h of deprivation or control, perfused with 0.9% phosphate buffered saline and 4% paraformaldehyde, and brains processed for c-Fos expression.

### c-Fos immunohistochemistry and quantification

Following fixation in 4% paraformaldehyde, brains were cryoprotected in 20% glycerol in PBS and stored at 4°C. Brains were coronally sectioned with a microtome at 40 μm. Free-floating sections were stained using standard immunohistochemical techniques. Following 0.1 M PBS rinses, tissue was exposed to anti-c-Fos primary antibody (polyclonal guinea pig, 1:1000, Cat. No. 226005, Synaptic Systems, Gottingen, Germany, RRID:AB_2800522) in Triton-X and goat serum blocking solution and incubated overnight at room temperature with gentle agitation. The sections were then washed with PBS and a polyclonal goat anti-guinea pig secondary antibody (Alexa 488, 1:1000, Abcam, Cat. No. 150185, RRID:AB_2736871) applied for visualization.

### Regions of interest and circuit analyzes

For quantification of activity within regions of interest (ROI), images of c-Fos + cells were taken at 4x with an Olympus BH-2 fluorescence microscope. C-Fos + cells were counted using ImageJ software (Research Services Branch, National Institute of Mental Health, Bethesda, Maryland, United States) by an observer blind to the condition and cell density was calculated as the mean cell number per square millimeter. C-Fos + cells were identified by size and relative intensity against background determined visually, though cell staining intensity was not quantified. ROI’s included the Salience Network: agranular insular cortex (AI), prelimbic cortex (PL), medial dorsal thalamus (MDN), central nucleus of the amygdala (ceA) and the ventral striatum (*VS*), with at least 3 sections included for each ROI/mouse. For comparison, we also created a novel collection of areas known to be affected by developmental ethanol and relevant for many of the behaviors known to be impacted by ethanol exposure (e.g., Berman and Hannigan, 2000; Hamilton et al., 2017; Kozanian et al., 2018). These included: PL, infralimbic cortex (IL), basolateral amygdala (BLA) and CA1, CA3 and dentate gyrus (DG) subregions of the hippocampal formation.

ROI activity levels (c-Fos + cell count density) were compared across groups (saline-rested control, saline-deprived, ethanol-rested control, ethanol-deprived) with ANOVA. Functional connectivity within the networks was determined by calculating a correlation matrix of all ROI pairs (Perry et al., 2016; Junod et al., 2019). While functional connectivity using fMRI or electrophysiological data involves within animal correlation or coherence of time series activity across regions (Shibasaki, 2008; Kelly et al., 2012; Xu et al., 2022), functional connectivity assessment based on correlations of static interregional metabolic or immediate early gene activity across animals, as used here, has been shown to be a reliable assay across multiple paradigms (Teles et al., 2015; Zuloaga et al., 2015; Perry et al., 2016; Tanimizu et al., 2017; Junod et al., 2019; Moretti et al., 2022; Santos et al., 2023; Stefaniuk et al., 2023).

Correlation matrices for ROIs were visualized for each group using heatmaps and also classical multidimensional scaling (MDS), which facilitated comparisons of connectomes. For MDS, correlations between ROI pairs were subtracted from 1 for conversion to proximities. MDS recovered these proximities as visualizable distances in a low-dimensional coordinate space. Placement of ROIs near one another in MDS space reflected similarity in their responses. Dissimilar ROIs were positioned apart. The overall arrangement of all ROIs reflected the correlation structure of the connectome for each group. Similar results were found using parametric and non-parametric correlation coefficients; we show results based on parametric (Pearson) correlations.

Comparisons in overall network activity and connectivity between groups was performed with Hierarchical Cluster Analysis (HCA). For HCA of activity and connectome similarity under the four different conditions, standard HCA routines in SPSS were used. An agglomerative protocol was used to determine clustering and the distance between clusters was determined by squared-Euclidean distance. This analysis allows a quantitative assessment of similarity between network wide activity and between connectomes across the different groups.

### Statistical analyzes

Statistical comparisons between groups and treatments were performed with ANOVA and/or t-tests using Prism 9 software. Statistical significance was set at *p* < 0.05. HCA was performed using SPSS, v24. Multidimensional scaling was performed using MATLAB, v9.12.

## Results

**Figure 1:**
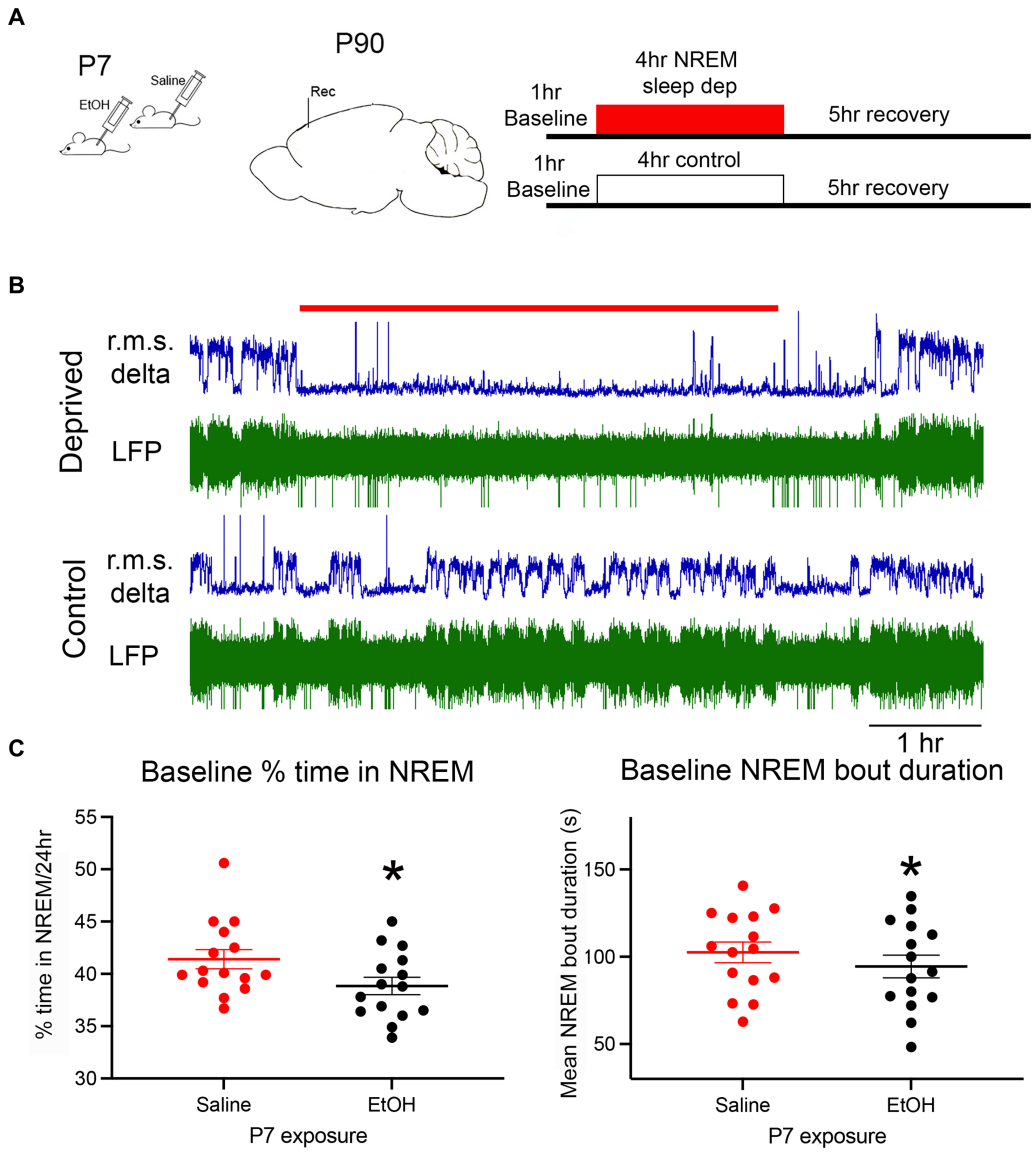
**(A)** The basic experimental protocol involved P7 injections of ethanol or saline. As adults, mice were implanted with electrodes for telemetry LFP recordings of cortical activity. Following baseline recordings, animals were exposed to an enrichment-based sleep deprivation protocol, or left alone, and then either allowed to recover or were immediately sacrificed for c-Fos immunohistochemistry. **(B)** Representative example of simultaneous recording from a sleep deprived mouse and a control littermate. R.M.S. delta amplitude is shown in blue above the raw recording. The red line denotes the 4 h sleep deprivation period. **(C)** Baseline NREM sleep measures show a significant reduction in the time spent in NREM sleep and NREM sleep bout duration in ethanol-exposed mice compared to saline controls as previously reported. Asterisks denote significant difference between saline and ethanol.

Baseline NREM sleep was recorded over a 24 h period prior to onset of the sleep deprivation protocol (Figure 1A). Figure 1B shows typical recordings of cortical LFP in a sleep deprived and control animal. Minimal large amplitude delta activity occurred during the sleep deprivation period. As previously reported (Wilson et al., 2016, 2023), based on the 24 h recordings developmental ethanol exposure resulted in significantly less time in NREM sleep [*t*-test, *t*(14) = 2.82, *p* = 0.007] and significantly shorter NREM sleep bouts [*t*(14) = 1.99, *p* = 0.033] indicative of NREM sleep fragmentation (Figure 1C).

### Sleep homeostasis

**Figure 2:**
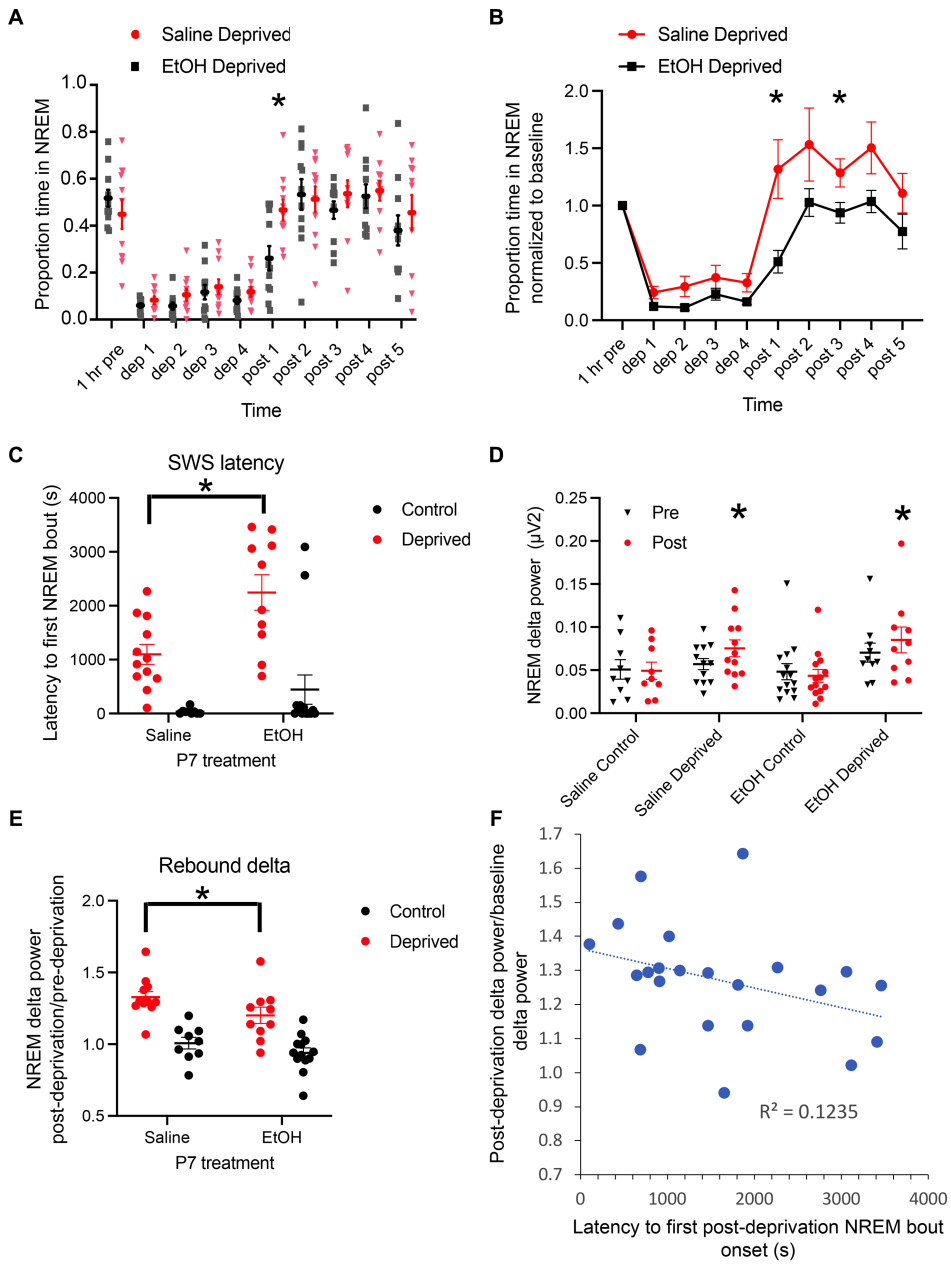
**(A)** Proportion of time spent in NREM sleep/h in saline- and ethanol-exposed mice during and after 4 h of sleep deprivation. While saline-exposed mice showed a rebound in proportion of time in NREM sleep after the end of deprivation, ethanol-exposed mice showed a delayed return to baseline. Asterisk signifies significant difference between ethanol and saline at 1 h post deprivation termination. **(B)** Proportion of time spent in NREM sleep, normalized to baseline in saline- and ethanol-exposed mice during and after 4 h of sleep deprivation. As in **A**, while saline-exposed mice showed a rebound in proportion of time in NREM sleep after the end of deprivation, ethanol-exposed mice showed a delayed return to baseline, with no rebound. Asterisks signify significant difference between ethanol and saline. **(C)** Latency between the end of sleep deprivation the onset of the first NREM sleep bout. Deprived saline-exposed mice showed a significantly shorter latency than deprived ethanol-exposed mice. **(D)** Raw NREM delta power pre- and post-deprivation or control manipulation across all four groups. There was no significant difference in baseline (pre) NREM delta power across groups. Sleep deprivation significantly enhanced NREM dela power in both P7 saline- and ethanol-exposed mice. Asterisks denote significant difference between pre- and post-deprivation delta power within groups. **(E)** Post-deprivation (or control) NREM sleep delta power expressed as a proportion of baseline NREM sleep delta power showed a strong increase in saline-exposed mice and this post-deprivation increase was significantly reduced in ethanol-exposed mice. Asterisks denote significant difference between saline and ethanol. **(F)** Across all animals regardless of P7 exposure, the latency to first NREM sleep bout was negatively correlated with post-deprivation NREM sleep delta power.

During the 1 h baseline prior to onset of sleep deprivation, behavioral activity within the home cage did not significantly differ between the four conditions (P7 exposure X treatment ANOVA, both main effects and interaction *p* > 0.05). Similarly, although over a 24 h period NREM sleep is reduced in P7 ethanol-exposed mice (Figure 1), there was no significant difference in proportion time in NREM sleep during the 1 h of undisturbed baseline prior to sleep deprivation onset (Figure 2A). Thus, immediately prior to sleep deprivation, the mice were in relatively comparable states. The sleep deprivation protocol was similarly effective in both saline (*n* = 10 rested control and 11 deprived) and ethanol-exposed (*n* = 13 rested control and 12 deprived) mice, with non-deprived, rested, controls spending about 45% of the 4 h hour period in NREM sleep (saline mean = 47.7 ± 3.9%, ethanol mean 42.7 ± 5.1%) and deprived animals spending about 12% in NREM sleep (saline mean = 13.3 ± 2.9%, ethanol mean 9.8 ± 1.5%; P7 exposure X treatment ANOVA, main effect of deprivation/control treatment *F*(1, 43) = 426.5, *p* < 0.0001, no significant effect of P7 exposure F(1, 43) = 2.175, *p* = 0.147). However, there was a significant interaction between P7 exposure X treatment suggesting that the deprivation protocol was slightly more effective in ethanol-exposed mice than saline-exposed mice [Figure 2B; *F*(1,43) = 4.146, *p* = 0.0479], potentially consistent with a decrease in sleep pressure in developmental ethanol-exposed mice. In order to determine whether there was a sex difference in sleep rebound between saline- and ethanol-exposed mice, the normalized time spent in NREM sleep during the first hour post deprivation was compared for the sleep deprived animals with a 2-way ANOVA (P7 treatment X sex). While there was a main effect of P7 treatment [*F*(1,18) = 9.99, *p* = 0.005] there was no main effect of sex [*F*(1,18) = 0.81, *p* = 0.38] nor a treatment X sex interaction [*F*(1,18) = 2.37, *p* = 0.14]. Saline exposed males (mean normalized time in NREM = 1.67 ± 0.49) and females (mean = 1.02 ± 0.20) showed more NREM during the first hour post-deprivation than male (mean = 0.42 ± 0.17) and female (mean = 0.59 ± 0.12) ethanol-exposed mice.

The NREM sleep deprivation and recovery data are presented both as raw proportion of time in NREM for each hour (Figure 2A), and as normalized within animals to the pre-deprivation 1 h baseline NREM sleep proportion (Figure 2B). Both analyzes show similar effects. Following sleep deprivation, saline-exposed mice displayed a strong rebound in NREM sleep compared to ethanol-exposed mice. A repeated measures ANOVA (P7 treatment X group) of the raw proportion time spent/h in NREM sleep revealed that saline-exposed mice spent significantly more time in NREM sleep following the end of the deprivation protocol than ethanol-exposed mice [main effect of exposure, *F*(3,40) = 9.78, *p* < 0.0001; main effect of time, *F*(5.1,203.6) = 20.45, *p* < 0.0001; time X treatment interaction *F*(27,360) = 9.00, *p* = 0.0001]. Post-hoc Fisher tests revealed no difference in time spent in NREM sleep between P7 saline- and P7 ethanol-exposed mice, and that P7 saline-exposed mice spent significantly more time in NREM sleep during the 1 h post deprivation than P7 ethanol exposed mice. Using NREM sleep proportion normalized within animals to the pre-NREM deprivation baseline, a selective comparison of the P7 saline and P7 ethanol NREM deprived mice showed same effect (Figure 2B) with P7 saline mice expressing a clear NREM rebound post deprivation (P7 exposure X time, main effect of exposure, *F*(1,20) = 7.814, *p* = 0.011; main effect of time, *F*(2.28,53.62) = 31.65, *p* < 0.0001; time X treatment interaction *F*(9,180) = 2.126, *p* = 0.029). For example, while saline-exposed mice rebounded to nearly 150% of pre-deprivation NREM sleep levels during the first hour post-deprivation, ethanol-exposed mice stayed below pre-deprivation levels, only returning to baseline during the second post-deprivation hour and never showing a detectable rebound effect in time spent in NREM sleep (Figure 2B). This differential response is also evident in the latency to the first NREM sleep bout following termination of the deprivation. Ethanol mice had a significantly longer latency to NREM sleep than saline-exposed mice (P7 exposure X deprivation treatment, main effect of deprivation treatment, *F*(1,41) = 33.01, *p* < 0.0001; main effect of P7 exposure, *F*(1,40) = 5.08, p = 0.029; no significant interaction F(1,41) = 2.21, *p* = 0.144). Post-hoc tests revealed that ethanol deprived mice had a significantly longer latency to first NREM bout than saline deprived mice (Figure 2C).

In addition to time spent in NREM sleep, we also examined slow-wave activity (delta band power, Figure 2D). A one-way ANOVA showed no significant difference in baseline NREM delta power between the four groups (saline-control, saline-deprived, ethanol-control and ethanol-deprived) during the 1 h before sleep deprivation or control [*F*(3,41) = 1.02, *p* = 0.393]. A 2 × 4 repeated measures ANOVA (pre/post X group) showed no main effect of group [*F*(3,40) = 1.597, *p* = 0.20 N.S.], but a significant interaction between group and time [pre and post; *F*(3,40) = 11.98, *p* < 0.001]. Post-hoc tests revealed no significant change between pre- and post- control condition NREM delta power, but a significant increase in delta power post-sleep deprivation in both P7 saline-exposed (*p* < 0.0001) and ethanol-exposed mice (*p* = 0.002). Within animal normalization of NREM delta power to baseline in the deprived animals confirmed a significant enhancement post-deprivation in saline-exposed and ethanol-exposed animals (Figure 2E; normalized post deprivation delta power, 2 × 2 ANOVA exposure X deprivation treatment, main effect of deprivation treatment, *F*(1,40) = 45.43, *p* < 0.0001; main effect of P7 exposure, *F*(1,40) = 5.087, *p* = 0.029; no significant interaction, *F*(1,40) = 0.47, *p* = 0.499). Post-hoc tests revealed that ethanol deprived mice had a significantly reduced slow-wave activity rebound compared to saline deprived mice (Figure 2E). Across all deprived animals (Figure 2F), the latency to express a rebound NREM sleep bout was significantly correlated with slow-wave activity rebound (*r* = −0.35, *p* = 0.05) as previously reported for measures of sleep pressure (Borbely et al., 1981; Brunner et al., 1990). Together, these results are consistent with a reduced sleep pressure in P7 ethanol-exposed mice.

### Sleep-dependent activity and functional connectome

**Figure 3:**
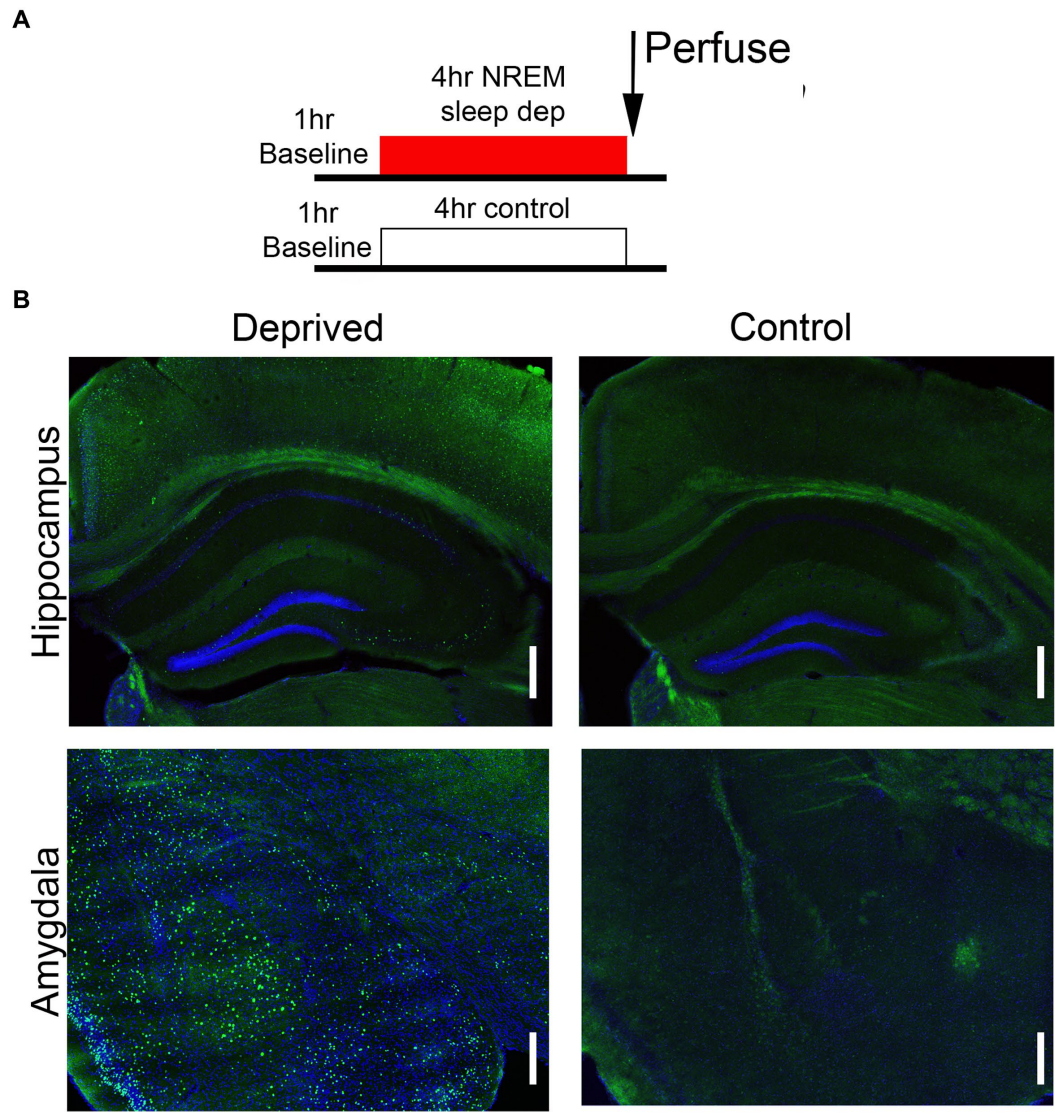
**(A)** Schematic of the protocol used for c-Fos assessment of circuit function following sleep deprivation. **(B)** Representative examples of c-Fos expression in amygdala and hippocampal formation following either 4 h deprivation or control. Sleep deprivation enhanced the density of c-Fos + cells in these and other brain regions. Scale bars are 500 μ.

**Figure 4:**
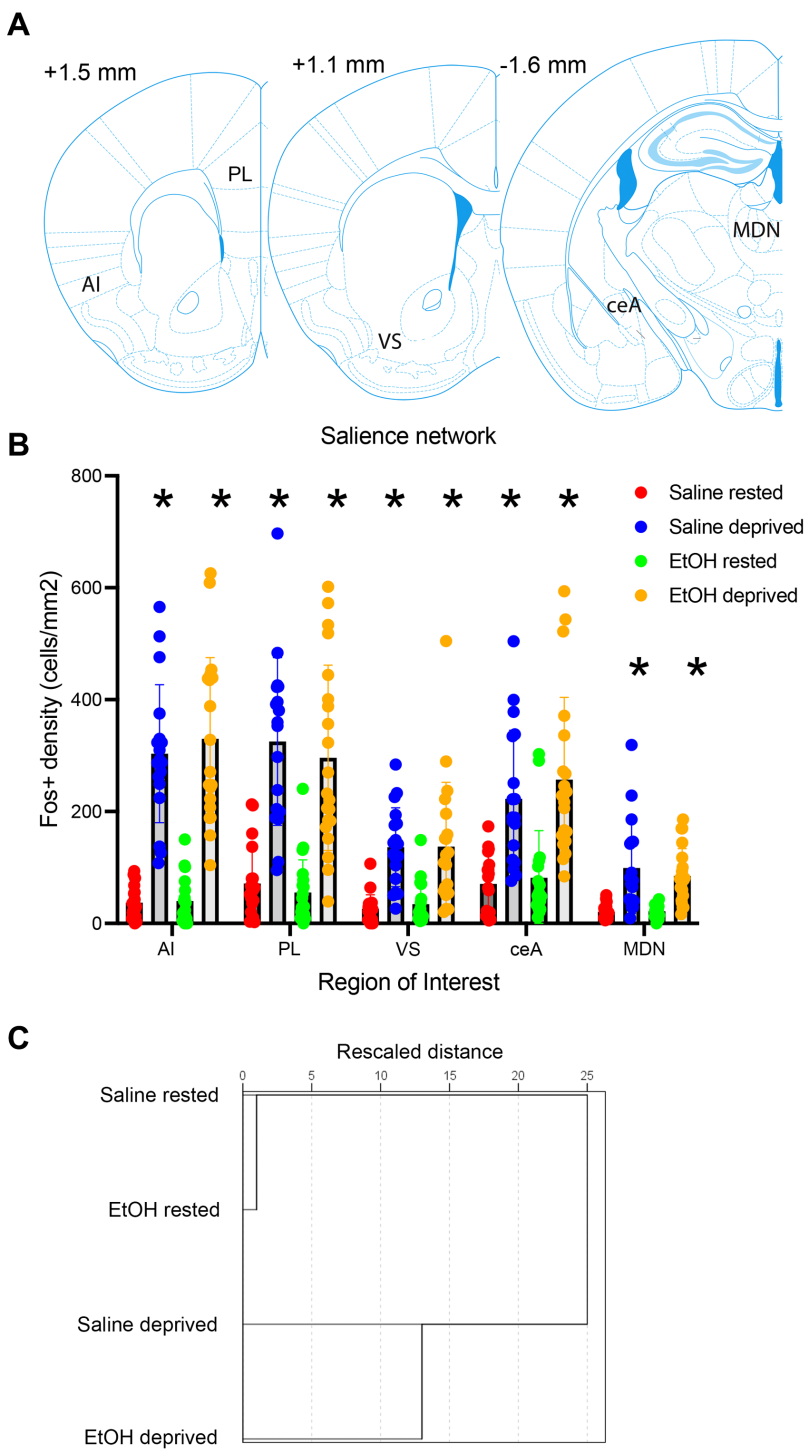
**(A)** Locations of the Salience network ROI’s used for c-Fos quantification. **(B)** c-Fos + density in each ROI in saline- and ethanol-exposed mice in the sleep deprivation or control (rested) condition. Sleep deprivation enhanced c-Fos + density in all ROI’s compared to controls regardless of developmental exposure. Asterisks signify significant difference between the deprived cell density compared to the rested cell density within that region. **(C)** HCA of c-Fos + density patterns in each group. This analysis reveals that rested animals showed more similar patterns of activity regardless of developmental exposure, while sleep deprived saline- and ethanol-exposed mice formed a separate cluster, suggesting no interaction between early exposure and sleep deprivation.

Activity within the Salience network during the environmental enrichment-induced sleep deprivation was assessed in animals sacrificed immediately after the 4 h deprivation period (Figure 3A) and analyzed at both the ROI and functional connectivity levels (saline rested control, *n* = 19; saline deprived, *n* = 18; ethanol rested control, *n* = 19; ethanol deprived, *n* = 21). C-Fos + cell density was enhanced in sleep-deprived mice compared to rested controls (Figure 3B). Quantitative analyzes of this effect demonstrated that enrichment-induced sleep deprivation significantly enhanced activity in all Salience network ROI’s (Figure 4A) in both saline- and ethanol-exposed mice (Figure 4B). A 5 × 4, ROI x Group ANOVA revealed a significant main effect of ROI [*F*(4,361) = 30.08, *p* < 0.0001], significant main effect of group [*F*(3, 361) = 108.8, *p* < 0.0001] and a significant ROI X group interaction [*F*(12, 361) = 6.079, *p* < 0.0001]. Post-hoc tests revealed significantly higher Fos + cell density in all ROI’s of deprived animals compared to controls, regardless of P7 exposure, though the effect was less pronounced in MDN and *VS* than in other Salience network ROI’s. In order to compare overall network activity between groups, HCA was performed on Fos + cell densities across the Salience network. As shown in Figure 4C, Salience network ROI activity patterns were most similar for the saline- and ethanol-exposed rested mice, with the saline- and ethanol-exposed sleep deprived mice forming a separate cluster. This suggests that Salience network ROI activity can distinguish between rested and sleep deprived animals, but is not sensitive to P7 ethanol exposure.

**Figure 5:**
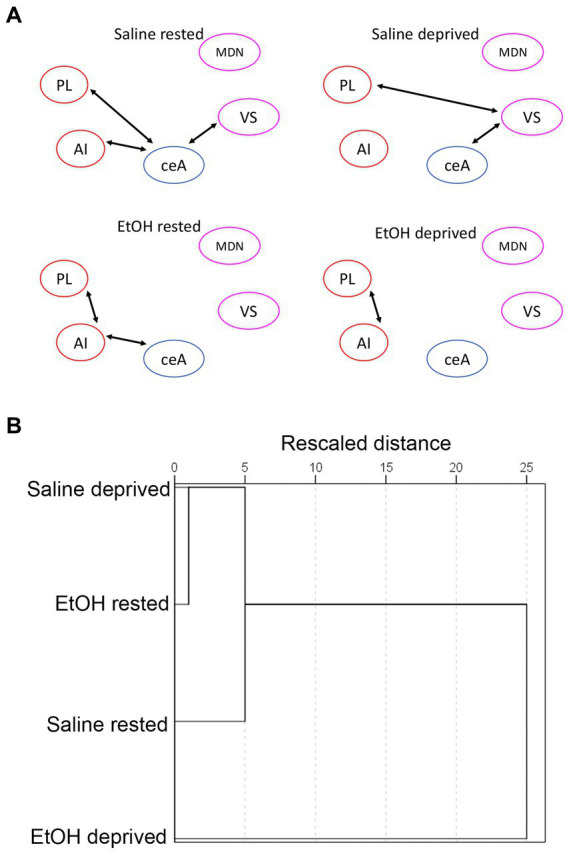
**(A)** Salience network functional connectivity based on developmental exposure and sleep condition. Arrows between ROIs signify a statistically significant correlation in c-Fos + cell densities (i.e., significant functional connectivity). Both sleep deprivation and ethanol exposure reduced functional connectivity from that observed in rested saline mice. **(B)** HCA of bivariate correlation matrixes in each group. Rested, ethanol exposed mice showed a salience network connectivity pattern that most closely matched that of sleep-deprived saline-exposed mice. Sleep-deprived, ethanol-exposed mice showed a connectivity pattern most distinct from all other groups.

**Figure 6:**
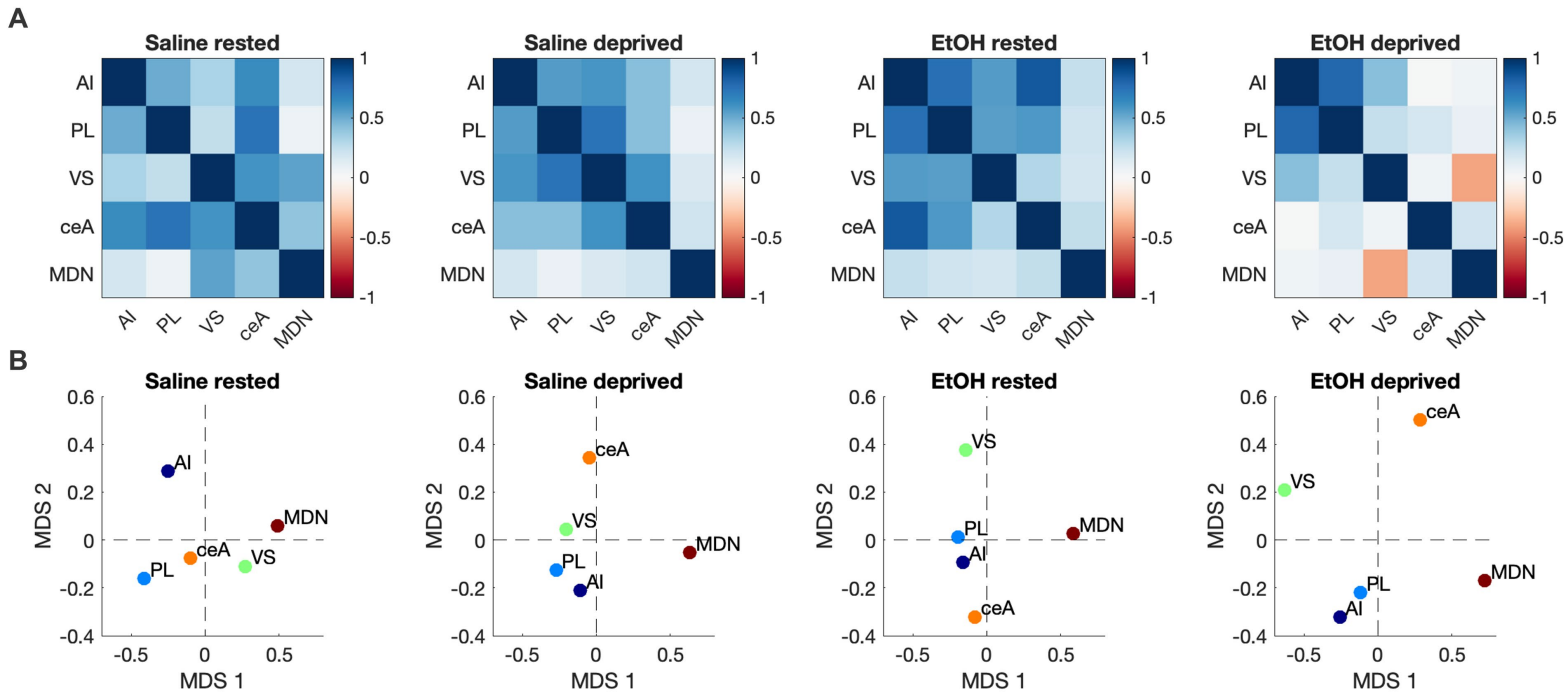
**(A)** Heatmaps showing pairwise correlations between ROIs in the Salience network for all groups. Color legend is Pearson’s *r*. **(B)** Two-dimensional plots showing Salience network correlation structures recovered by MDS for all groups. Correlation patterns and associated ROI arrangements in MDS space both suggest the Salience network connectome of rested, ethanol exposed mice was most similar to that of sleep-deprived saline-exposed mice.

We then assessed functional connectivity within the Salience network in these same animals. As described elsewhere (Perry et al., 2016; Junod et al., 2019) functional connectivity was determined by bivariate correlations in activity between all ROI’s in the network. Figure 5A displays the pathways showing statistically significant activity correlations (i.e., functional connectivity) in each group. In each group, the PL shows a significant functional connection with at least one other Salience network ROI, with the rested Saline mice showing the greatest overall connectivity. Both sleep deprivation and P7 ethanol exposure reduced the number of significant connections, with ethanol-deprived mice limited to a single remaining connection. In order to quantitatively compare the functional connectomes between groups, HCA was performed on the full correlation matrix for each group whether the individual correlations were significant or not (Figure 5B). This HCA revealed that the Salience network functional connectome of rested-ethanol exposed mice was most similar to that of sleep-deprived saline-exposed mice. This trend was also apparent in visual representations of the Salience network connectome provided by correlation heatmaps (Figure 6A) and MDS (Figure 6B), where rested-ethanol exposed and sleep-deprived saline-exposed mice showed similar ROI correlation structures. Thus, even when given the opportunity for *ad lib* sleep, the Salience network of developmental ethanol-exposed mice displays functional connectivity similar to that of sleep deprived saline-exposed mice. Sleep deprivation of ethanol-exposed mice results in an outlier functional connectivity pattern, as is evident both the HCA analysis and in the number of significant connections shown in Figure 5A.

**Figure 7:**
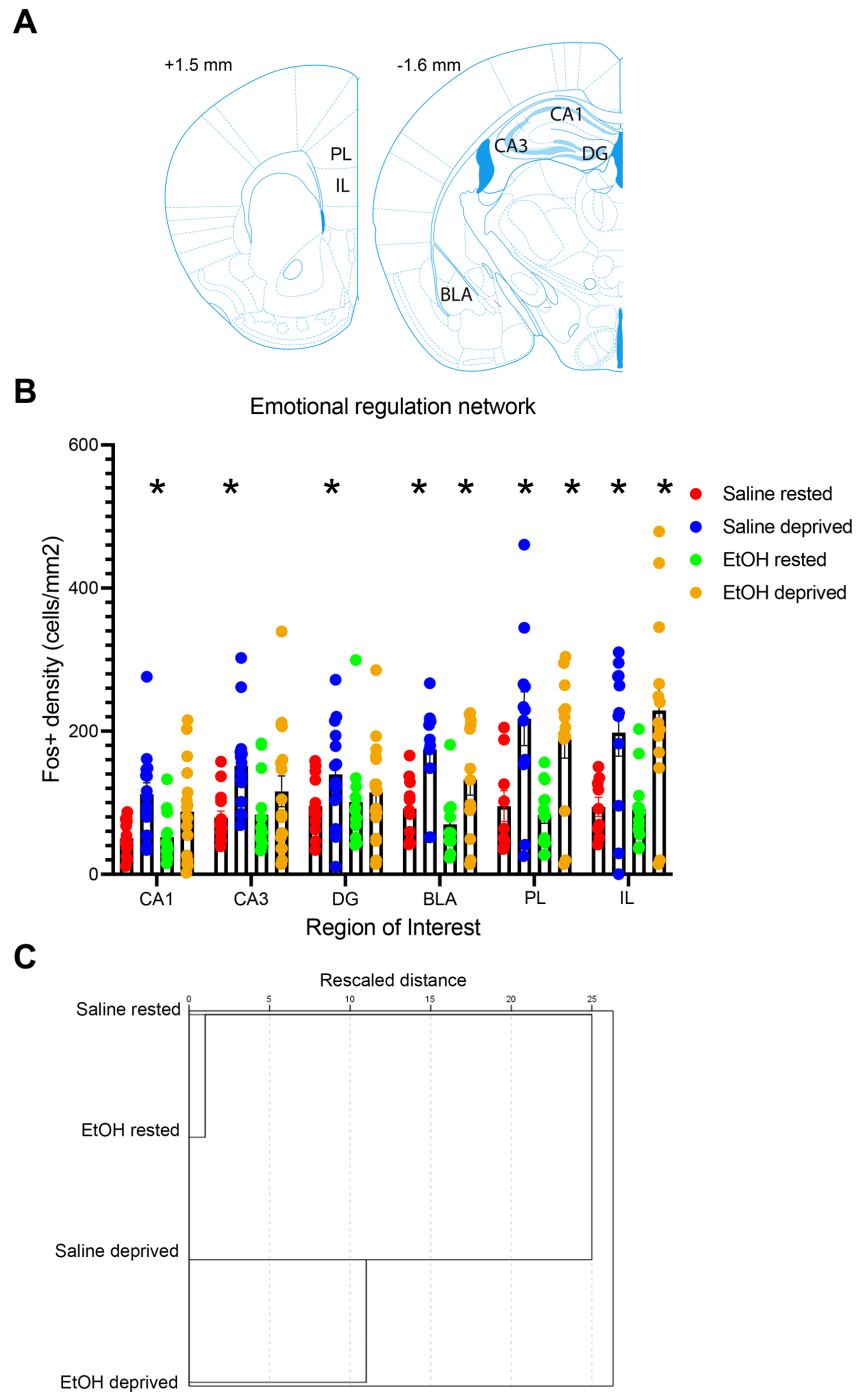
**(A)** Locations of the control network ROI’s used for c-Fos quantification. **(B)** c-Fos + density in each ROI in saline- and ethanol-exposed mice in the sleep deprivation or control (rested) condition. Sleep deprivation significantly increased c-Fos + density in all ROI’s compared to rested animals in P7 saline-treated mice. However, in P7 EtOH-treated mice this deprivation-induced increase was only observed in regions outside of the hippocampal formation (DG, CA3, CA1). Asterisks signify significant difference between the deprived cell density compared to the rested cell density within that region and developmental exposure. **(C)** HCA of c-Fos + density patterns in the control network for each group. As in the Salience network, HCA reveals that rested animals showed more similar patterns of activity regardless of developmental exposure, while sleep deprived saline- and ethanol-exposed mice formed a separate cluster, suggesting no interaction between early exposure and sleep deprivation.

In order to test the specificity of these effects to the Salience network, a novel network was constructed of ROI’s known to underlie many of the cognitive and emotional behaviors impacted by developmental ethanol exposure. ROIs included in this artificial network were the PL, IL, BLA, and hippocampal CA1, CA3 and DG (Figure 7A). As shown in Figure 7B, similarly to the Salience network, all ROI’s in saline-exposed mice showed enhanced activity in response to enrichment-induced sleep deprivation. However, in ethanol-exposed mice, while medial prefrontal cortex and BLA showed sleep deprivation-induced increases in activity, hippocampal subregions did not (Figure 7B). A 6 × 4, ROI x Group ANOVA revealed a significant main effect of ROI [*F* (3.47, 164.4) = 12.74, *p* < 0.0001], a significant main effect of group [*F* (3, 56) = 10.06, *p* < 0.0001] and a significant ROI X group interaction [*F* (15, 237) = 2.512, *p* = 0.002]. HCA analysis of ROI activity across groups showed that, as with the Salience network, activity patterns in this control network were most similar for the saline- and ethanol-exposed rested mice, with the saline- and ethanol-exposed sleep deprived mice forming a separate cluster.

**Figure 8:**
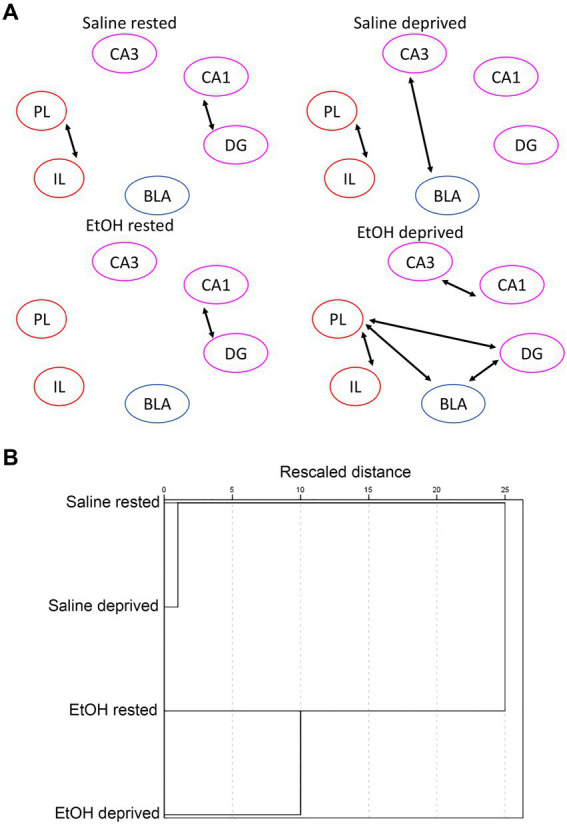
**(A)** Control network functional connectivity based on developmental exposure and sleep condition. Arrows between ROI’s signify a statistically significant correlation in c-Fos + cell densities (i.e., significant functional connectivity). In contrast to the Salience network the combination of sleep deprivation and ethanol exposure enhanced functional connectivity from that observed in rested saline mice. **(B)** HCA of control network bivariate correlation matrixes in each group. In contrast to the Salience network, HCA revealed similar functional connectivity patterns for saline-exposed mice, regardless of sleep deprivation status, while ethanol-exposed deprived and rested mice formed a separate cluster. This suggests that functional connectivity in this network is sensitive to early exposure but not to sleep/rest state.

**Figure 9:**
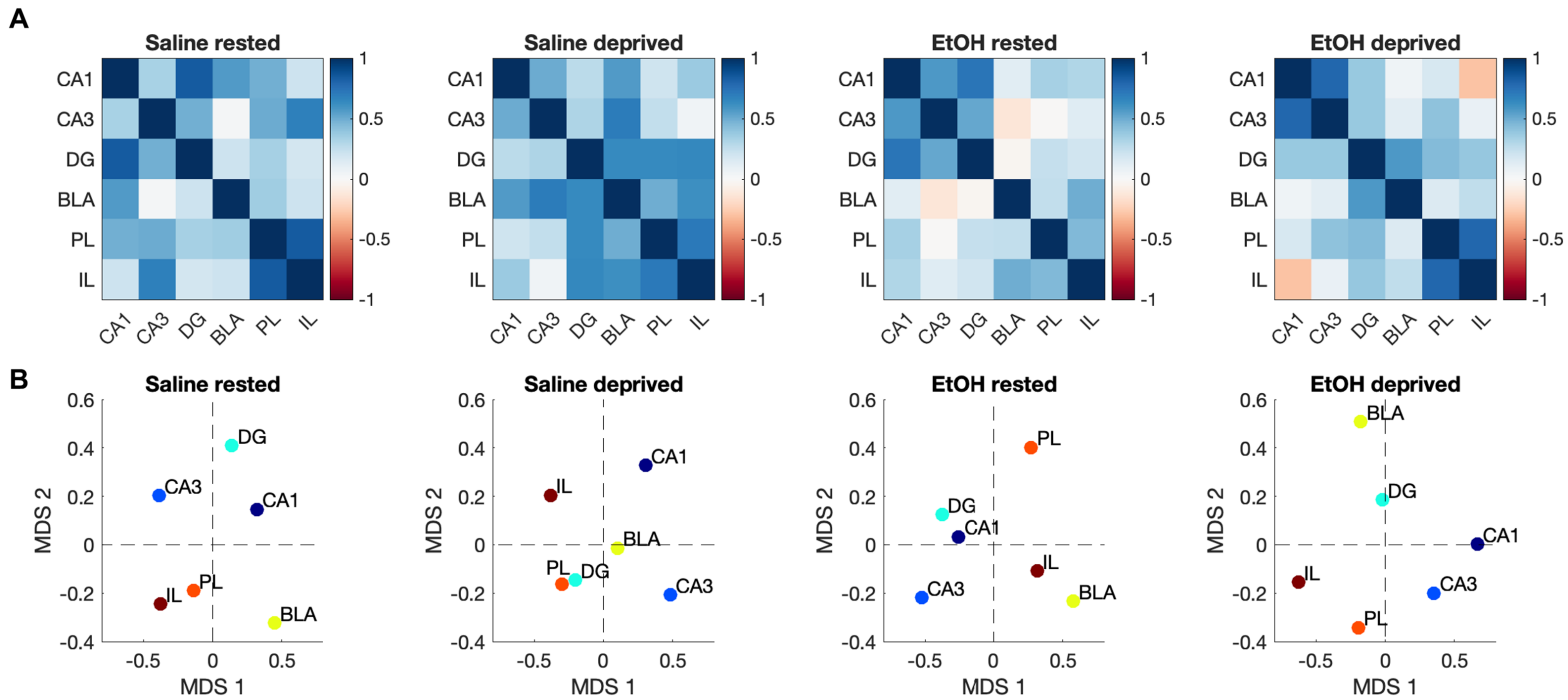
**(A)** Heatmaps showing pairwise correlations between ROIs in the control network for all groups. Color legend is Pearson’s *r*. **(B)** Two-dimensional plots showing control network correlation structures recovered by MDS for all groups. Unlike the Salience network, the control network connectomes for rested, ethanol exposed and sleep-deprived saline-exposed mice displayed dissimilarities.

In contrast to similarities between this control network and the Salience network in terms of ROI activity in response to ethanol and sleep deprivation, functional connectivity responses were markedly different between the two networks. A shown in Figure 8A, sleep-deprived ethanol-exposed mice showed enhanced functional connectivity within this network compared to other conditions. This contrasts with the decreased functional connectivity observed in the Salience network induced by ethanol exposure and by sleep deprivation. Furthermore, HCA analysis (Figure 8B) revealed that, as with ROI activity levels, functional connectivity was most similar between saline exposed mice, regardless of sleep condition, with ethanol-exposed mice forming a separate cluster. Thus, functional connectivity within this network was much more sensitive to developmental exposure and much less sensitive to recent sleep conditions. Finally, heatmap (Figure 9A) and MDS (Figure 9B) connectome mappings for the control network revealed that rested-ethanol exposed and sleep-deprived saline-exposed mice showed markedly different ROI correlation structures, which differed from the ROI association apparent between these groups in the Salience network.

## Discussion

The present results demonstrate three major impacts of developmental ethanol on sleep and begin to provide insight into how sleep disturbance in this model could impact cognition and emotional regulation in adults. First, as previously reported (Wilson et al., 2016, 2023), P7 binge ethanol exposure resulted in a reduction and fragmentation of NREM sleep in adult mice (Figure 1). Second, P7 ethanol impaired sleep homeostasis in adults, effectively eliminating rebound NREM sleep and slightly reducing rebound slow-wave activity following sleep restriction. Third, functional connectivity in the Salience network, a network involved in responding to external and internal events such as rewards and punishments (Seeley, 2019) as well as processing motivational state and emotion (Schimmelpfennig et al., 2023) is disrupted by developmental ethanol such that even when rested, ethanol-exposed mice display the functional equivalent of sleep-deprived Salience network functional connectivity. This modification of Salience network connectivity was not observed in a control network.

Sleep homeostasis refers to process of regulating sleep–wake cycles by balancing the amount and quality of sleep over time. This process reflects the increase in sleep pressure over waking periods, at least in part due to adenosine build-up (Porkka-Heiskanen et al., 1997) from astrocytic release (Halassa et al., 2009; Artiushin and Sehgal, 2020), and its depletion during sleep. Thus, following a period of sustained wakefulness, sleep duration and slow-wave activity are increased (Huber et al., 2007; Vyazovskiy et al., 2011; Tononi and Cirelli, 2014). Changes in slow-wave activity have also been hypothesized to reflect synaptic potentiation induced by experiences occurring during waking and synaptic strength renormalization during sleep which allows re-setting of synapses for new learning upon re-awakening (Tononi and Cirelli, 2014). Importantly, disrupted sleep homeostasis has been linked to impaired cognitive and emotional function in humans and animal models (Armitage, 2007; Halassa et al., 2009; Boly et al., 2017). The results here suggest that reduced NREM sleep and NREM sleep fragmentation induced by developmental ethanol may in part be due to reduced sleep pressure or sensing thereof. Furthermore, in addition to the direct impacts of sleep fragmentation induced by developmental ethanol on cognition, emotion and behavior (Wilson et al., 2016; Lewin et al., 2018), the additional impairment in sleep homeostasis could further disrupt normal sleep cycles. We did not examine REM sleep nor microsleeps in this study, both of which could be impacted by developmental ethanol exposure. Sleep deprivation in both rodents and humans can result in microsleeps that can last less than the 14 s bins used here to quantify NREM/wake states (Friedman et al., 1979; Makeig et al., 2000; Goel et al., 2009; Poudel et al., 2014) and can help reduce sleep pressure (Dement, 1972). It is unknown whether developmental ethanol exposure impacts microsleeps but this should be examined given the impact microsleeps have on attention and cognition (Makeig et al., 2000; Goel et al., 2009).

The mechanisms by which developmental ethanol exposure impacts NREM sleep homeostasis are unknown, however, at least two factors may contribute. First, as noted above, astrocytes play an important role in sleep homeostasis and astrocytes are strongly affected by developmental ethanol exposure in both number and function (Valles et al., 1996; Rubert et al., 2006; Guizzetti et al., 2014; Trindade et al., 2016). It should also be noted that acute alcohol can also disrupt sleep homeostasis (Thakkar et al., 2015). Thus, loss of astrocytes or normal astrocytic function induced by developmental ethanol exposure may contribute to the observed decrease in sleep homeostasis by reducing adenosine build-up during waking. A second factor may relate to the observed impairment in synaptic potentiation induced by developmental ethanol (Swartzwelder et al., 1988; Izumi et al., 2005; Sadrian et al., 2012; Subbanna et al., 2013)–though see (Fontaine et al., 2016) for caveats. If increases in slow-wave activity during waking reflect synaptic potentiation which is then de-potentiated during subsequent sleep (Tononi and Cirelli, 2014), any impairment in experience-dependent potentiation and/or synaptic depression may be expected to reduce slow-wave activity signatures of sleep homeostasis as seen here.

In addition to disruption of NREM sleep homeostasis, adult Salience network function was also modified. Activity within all network ROI’s was enhanced by sleep deprivation, which most likely reflects both maintained waking and the sensory stimulation used to induce it given the reported function of this network (Seeley, 2019). There was no effect of developmental ethanol exposure on ROI activity levels in either control or deprived mice. However, in contrast to ROI activity, Salience network functional connectivity was modified by developmental ethanol exposure and its interaction with sleep deprivation. Functional connectivity in diverse networks has been shown in both humans and animal models to be sensitive to diverse adverse events and pathologies including early life adversity (Scheinost et al., 2017; Jamieson et al., 2020) and FASD (Long et al., 2018; Roos et al., 2021). Network changes can include both abnormal increases or decreases in connectivity (Hanson et al., 2018; Johnson et al., 2018; Hanson et al., 2019). In the case of the Salience network, insomnia in humans is associated with decreases in intra-network connectivity (Khazaie et al., 2017) and increases in connectivity between Salience network and other networks such as the default mode network (Khazaie et al., 2017; Li et al., 2018; Chee and Zhou, 2019).

Here, we found a decrease in functional connectivity within the Salience network of adults exposed to developmental ethanol which mimicked the connectivity observed in saline-exposed mice that were intentionally sleep deprived. Thus, the decrease and fragmentation of NREM sleep induced by developmental ethanol in adults that were allowed *ad lib* sleep maintained a sleep-deprived functional connectome in the Salience network. Imposed sleep deprivation in developmental ethanol-exposed mice further disrupted network connectivity. Given the role of the Salience network in monitoring salient, homeostatically relevant events in the environment and internal state and its role in switching between networks critical for planning and execution of appropriate action, disruption due to sleep deprivation or developmental ethanol exposure would be expected to be highly relevant for the diversity of behavioral, cognitive and emotional sequela of FASD. These results may be useful in guiding more detailed analyzes of how different nodes and connections in this network contribute to cognitive, emotional and behavioral outcomes after developmental ethanol exposure.

The breakdown of functional connectivity in the Salience network after developmental ethanol was not a general phenomenon of brain networks. In fact, analysis of a control network composed of regions known to be impacted by, and behaviorally relevant for, outcomes associated with developmental ethanol showed weak sensitivity to early ethanol. For example, as with the Salience network, sleep deprivation enhanced activity in most ROI’s of this control network in both saline- and ethanol-exposed mice, though activity in the hippocampal formation was not enhanced in sleep-deprived ethanol-exposed mice. Furthermore, saline-rested and saline-sleep-deprived animals showed the most similar functional connectomes with ethanol-exposed animals falling in a separate cluster, suggesting no interaction between early life exposure and sleep on this control network. While other networks may show effects similar to the Salience network, this control analysis demonstrates that whole brain function is not universally impacted.

The mechanisms of changes in Salience network functional connectivity are not known. Network connectivity can be directly modified by changes in excitation/inhibition balance, synaptic plasticity, neuromodulators, changes in neural and/or glial cell structure or function, hormonal fluctuations, and a variety of other factors (Stephan et al., 2006; Gujar et al., 2010; Liston et al., 2011; Fields et al., 2015; Hall et al., 2015). Developmental ethanol impacts all of these factors including changes in excitation/inhibition balance (Sadrian et al., 2013), synaptic plasticity (Swartzwelder et al., 1988; Izumi et al., 2005; Sadrian et al., 2012; Subbanna et al., 2013), neuromodulators (Weinberg et al., 2008; Olateju et al., 2017; Heroux et al., 2019; Milbocker and Klintsova, 2021; Smiley et al., 2021), changes in the density, structure or function of both neurons (Ikonomidou et al., 2000; Wozniak et al., 2004; Saito et al., 2019; Smiley et al., 2019) and glial cells (Guizzetti et al., 2014). Thus, a variety of early ethanol consequences could contribute to the changes in Salience network functional connectivity shown here.

In summary, developmental ethanol exposure led to reduced NREM sleep and disrupted NREM sleep homeostasis in adult mice. This reduction in NREM sleep quality was associated with altered connectivity within the Salience network, which plays a crucial role in cognitive and emotional processing. Furthermore, the impairment in NREM sleep homeostasis and altered connectivity in the Salience network may exacerbate the negative impacts of developmental ethanol exposure on cognition and emotional regulation in adults. The mechanisms underlying these effects are not fully understood, but abnormal or altered astrocytic function induced by developmental ethanol exposure and impairment in synaptic potentiation are possible contributors. This impact on NREM sleep is an insidious daily remnant of developmental ethanol exposure. Targeting sleep may be a useful avenue to ameliorate the long last cognitive and emotional impacts of fetal alcohol (Ehlers et al., 2020).

## Data availability statement

The raw data supporting the conclusions of this article will be made available by the authors, without undue reservation.

## Ethics statement

The animal study was approved by Nathan Kline Institute Institutional Animal Care and Use Committee. The study was conducted in accordance with the local legislation and institutional requirements.

## Author contributions

PS: Investigation, Writing – review & editing. AK: Investigation, Writing – review & editing. GF: Investigation, Writing – review & editing. CW: Investigation, Writing – review & editing. RS: Writing – review & editing. CL: Writing – review & editing, Formal analysis. JS: Writing – review & editing, Funding acquisition. MS: Funding acquisition, Writing – review & editing. DW: Funding acquisition, Writing – review & editing, Conceptualization, Formal analysis, Investigation, Methodology, Project administration, Writing – original draft.
